# The role of the ethics expert in Spanish legislation on euthanasia and mental health

**DOI:** 10.1007/s40592-025-00228-3

**Published:** 2025-02-16

**Authors:** Sergio Ramos-Pozón

**Affiliations:** University of Barcelona, Barcelona, Spain

**Keywords:** Euthanasia, Mental health, Shared decision-making, Bioethics

## Abstract

This article examines the assessment of mental capacity in the context of euthanasia, particularly when requested by patients with mental illnesses. It proposes a holistic alternative approach to the traditional functional model, arguing that the latter is insufficient to capture the complexity of these patients’ decisions. Using approaches based on narrative, hermeneutic, and dialogical ethics, it offers an evaluation that considers the patient’s life story, values, and context. Shared decision-making and empathy are identified as fundamental components to ensure informed and consensual decisions, promoting an environment of respect and mutual understanding. The article reviews Spanish legislation on euthanasia, highlighting the need to include medical ethics experts in the Guarantee and Evaluation Commissions. These experts provide a comprehensive ethical perspective essential for addressing the ethical complexities in euthanasia requests and ensuring fair decisions that reflect the patient’s true will. It recommends reviewing and expanding current protocols, as well as including continuous ethics training to improve medical practice in this context. The conclusions suggest that an assessment of mental capacity based on ethical principles and an integrated narrative can significantly improve medical practice and decision-making in euthanasia, especially for these patients. Furthermore, the inclusion of ethics experts in the commissions can provide a more humane and just perspective, ensuring that decisions respect the patient’s dignity and autonomy.

## Introduction

In Spain, euthanasia was decriminalized with Organic Law 3/2021 of March 24, making it the fourth European country to enact a law on this issue and the first to regulate an ‘ex ante’ procedure through prior control by the Guarantee Commission (Organic Law 3/2021). This law values patient autonomy and allows healthcare professionals to invoke conscientious objection, refusing to participate for reasons of conscience. However, the application of euthanasia in patients with mental health issues presents specific challenges that require particular attention to the patient’s mental capacity.

The legislation introduces two modalities of assisted dying: euthanasia and medically assisted suicide. Crucial are the roles of the “responsible physician,” who coordinates the patient’s information and assistance, and the “consulting physician,” a specialist in the patient’s pathologies who is not part of the responsible physician’s team. Generally, for euthanasia or assisted suicide to be applied, the patient must have mental capacity, suffer from a serious, chronic, and debilitating condition, or have a serious and incurable illness.

Since the law is recent, data is still scarce. The latest official data indicates that since its implementation, 651 euthanasia procedures have been performed, with oncological and neurological diseases being the most common (Ministerio de Sanidad, 2022). Little is known about euthanasia for mental health issues compared to other countries where it is decriminalized (Calati et al. 2021; Evenblij et al. 2019; Verhofstadt M., et al., 2017).

The applicability of euthanasia in patients with mental health issues generates controversy regarding its legitimacy. Several studies (Evenblij et al. 2019; Pronk et al. 2021; Levin et al. 2020; Verhofstadt et al. 2020; Stefanello 2022; Sulzer et al. 2016) indicate that psychiatrists who reject it argue that it is very difficult to measure unbearable suffering or assess the patient’s mental capacity. Physicians also acknowledge that they more readily accept a request for euthanasia due to a somatic illness than for a mental pathology. The discrepancies focus on the assessment of mental capacity, the level of suffering, the irremediability of the illness, self-determination, and the authenticity of the decision (Nicolini et al. 2020).

Organic Law 3/2021 defines mental capacity inversely as the “situation in which the patient lacks sufficient understanding and will to autonomously, fully, and effectively govern themselves, regardless of whether support measures for the exercise of their legal capacity exist or have been adopted” (Organic Law 3/2021, art. 3.).

It is worth noting that not all vulnerabilities directly affect a person’s autonomy (Aguiar 2023). In the context of euthanasia and mental health, it is likely that vulnerabilities impacting decision-making—and therefore mental capacity—are associated with situations such as severe psychotic episodes, major depressive disorders, etc. These circumstances may affect the ability to process information, evaluate therapeutic alternatives, or make reasoned decisions consistent with one’s own values. In any case, analyzing these vulnerabilities requires not only tools like the MacCAT-T but also an ethical approach that promotes respect for and protection of the patient’s human rights. In this regard, we consider shared decision-making to be an effective strategy for mitigating vulnerabilities, as it fosters empathetic deliberation that incorporates the patient’s perspectives and values. This can reduce the risk of paternalistic or discriminatory decisions (Ramos et al. 2024).

Identifying vulnerabilities that affect autonomy demands an interdisciplinary approach encompassing medical, psychological, and ethical perspectives. In this sense, healthcare professionals and ethics experts could evaluate how the patient’s specific conditions influence decision-making. This would enable more reasoned, prudent, and deliberate choices. However, we believe that this analysis should consider the patient holistically and integrally, addressing their bio-psycho-social context. Furthermore, we contend that such an analysis prioritizes empathy and an understanding of the patient’s life story. By fostering shared decision-making, the care team can identify barriers to autonomy and work on specific strategies to overcome them. Examples might include providing information tailored to the individual’s cognitive abilities or strengthening support systems. This approach not only ensures more informed decisions but also promotes respect for the patient’s human rights, even in situations of extreme vulnerability.

In the realm of mental health, some studies (Vicens et al. 2021; Calcedo et al. 2020) conclude that many patients with severe mental disorders, such as schizophrenia or bipolar disorder, can responsibly decide about their health status. Therefore, there is no reason to dismiss a request for assisted dying on the grounds that the patient cannot decide.

We consider that patients with mental disorders who request euthanasia require a specific approach to assess their mental capacity due to the complexity and uniqueness of their experiences. Unlike somatic illnesses, mental disorders can uniquely affect the perception of suffering, the ability to assess quality of life, and the coherence of their decisions. However, this does not imply that all patients with mental disorders lack capacity; rather, their evaluation must go beyond traditional functional models, integrating emotional, ethical, and narrative aspects. This holistic approach allows for capturing how the patient’s values and emotions may influence their decision-making, ensuring a more just and respectful assessment of their autonomy.

In the context of euthanasia, especially in patients with mental disorders, integrating values and emotions into the assessment of mental capacity is ethically essential. These elements allow for capturing the subjective experience of suffering and the patient’s life story, offering a more humane and respectful evaluation of their autonomy and life project. Considering emotional and value aspects ensures more just decisions that align with the patient’s personal reality.

Another point of discrepancy among professionals is the issue of assessing suffering. Suffering can have various dimensions: medical, psycho-emotional, socio-environmental, and existential (Verhofstadt et al. 2021a, b; Stoll J., et al., 2021). Medical aspects include physical symptoms (fatigue, pain, problems with eating and drinking), cognitive symptoms (concentration problems, cognitive impairment), and psychiatric symptoms (loss of emotional control, suicidal thoughts, etc.). Psycho-emotional factors encompass the loss of autonomy and fear of future suffering. Socio-environmental dimensions include interpersonal experiences such as the loss of loved ones or psychological trauma, as well as difficulties in social relationships and economic and work support. Finally, existential suffering manifests as panic at continuing to live due to poor quality of life and lack of control over oneself (Verhofstadt et al. 2021a, b).

In this article, we will deeply analyze mental capacity, criticizing the traditional functional model and proposing a holistic evaluation that includes ethical and emotional aspects. Secondly, we will highlight the importance of shared decision-making in this care process. This will allow us to discuss the crucial role of empathy in this assessment, emphasizing how it improves support and informed decision-making. We will also propose ethical principles and a theoretical ethical foundation as the epistemological basis in the evaluation of mental capacity, integrating the patient’s life story and values. Finally, we will argue for the need to include medical ethics experts in this care process to address the ethical complexities of euthanasia requests from patients with mental illnesses.

## On mental capacity

Mental capacity to make decisions in the health field is fundamental to ensuring that patients can exercise their autonomy and make informed decisions about their health. In the context of euthanasia, this assessment ensures that the person can make this decision voluntarily, fully understands the implications and consequences of their request, is not influenced by their psychiatric symptoms, and that their decision is consistent with their values.

Organic Law 3/2021 requires a person to have mental capacity to receive euthanasia. The request must be an autonomous decision based on adequate information provided by the healthcare team. Euthanasia will not be applied if the responsible physician certifies that the patient is not in full possession of their faculties and cannot give their consent freely, voluntarily, and consciously.

Generally, mental capacity is considered to include the psychological aptitudes and skills necessary to make reasoned decisions about one’s health status. One of the most commonly used tools to assess such mental capacity is the MacArthur Competence Assessment Tool for Treatment (MacCAT-T) (Grisso, T., & Appelbaum, P.S., 1998). The MacCAT-T focuses on four key aspects: understanding, appreciation, reasoning, and choosing a decision. Understanding assesses the patient’s ability to comprehend information about their illness and treatment options. Appreciation measures whether the patient recognizes the relevance of this information to their situation. Reasoning examines the patient’s ability to compare options and foresee consequences. Finally, choosing a decision verifies whether the patient can clearly communicate their treatment preference.

Although it is a solid and widely accepted instrument, it has received some criticism. This article highlights the insufficiencies of the MacCAT-T and proposes an assessment of mental capacity that incorporates more human, ethical, and respectful aspects of the decisions of patients with mental illnesses. To integrate values and emotions into the assessment of decision-making capacity, methods such as narrative interviews can be used to explore the patient’s values. This would allow for exploring the patient’s life story, beliefs, and emotional contexts, offering a more qualitative and humane evaluation. In this way, we attempt to deeply understand the patient’s decisions beyond purely functional criteria. Alternative approaches from narrative, hermeneutic, and dialogical ethics are explored for a more comprehensive evaluation.

Banner and Szmukler (2013) argue that the procedural approach of the MacCAT-T aims to be normatively neutral; however, it does not capture the complexity of decisions in clinical practice. This interview ignores the underlying beliefs and values of the patient. To address all complexities, the authors recommend a holistic approach. This approach not only evaluates the decision-making process but also the coherence and validity of the patient’s beliefs and values in relation to their overall context. They propose the “principle of charity,” which involves assuming that the patient’s beliefs are consistent and correct. This requires attributing consistency and truthfulness to their beliefs and actions, seeking an interpretation that makes their behavior understandable and rational. Evaluating the content of beliefs and their relation to reality can offer a more complete and accurate assessment.

Narrative ethics emphasizes the importance of interpreting the patient’s life stories and fostering a hermeneutic dialogue in the evaluation of decision-making capacity. This approach allows for connecting with the patient’s subjective experience and, in turn, evaluating the coherence and authenticity of their decisions by integrating values and emotions. Thus, narrative ethics becomes a practical tool for a more comprehensive and respectful assessment of the patient’s autonomy.

Another author who also provides criticism is Kluge (2005), who considers that the notion of mental capacity must be integrated into a broader perspective than that of the traditional cognitive model. He argues that mental capacity should not only be assessed in terms of cognitive abilities but should also include emotional and value parameters. Kluge breaks down mental capacity into three areas: cognitive, emotional, and valuative. Firstly, cognitive capacity focuses on the individual’s rational abilities. Secondly, emotional capacity recognizes that humans interact in contexts that affect their decisions. Lastly, valuative capacity implies that decisions should be based on stable values over time.

Breden and Vollmann (2004) argue that the cognitive approach is based on a normative convention that attributes anthropological values without capturing the complexity of the decision-making process in real life. This model, centered on rational argumentation and logical consistency, does not always reflect how people make decisions, as these are often based on emotions, values, or intuitive factors.

Since everyday decisions often depend on subjective and contextual meanings, it is essential to broaden the theoretical assumptions of the MacCAT-T. Incorporating emotions, values, and personal constructs into the assessment allows for a more comprehensive and accurate understanding of the decision-making process. This involves interpreting cognitive aspects qualitatively, considering the patient’s value system. This better captures the importance of emotional, biographical, and contextual aspects in the assessment of mental capacity. Therefore, we advocate for extending the theoretical foundations of the MacCAT-T through alternative approaches from narrative, hermeneutic, and dialogical ethics.

As observed, it is essential to carefully examine the values, emotions, ethical principles, and type of care relationship that should be established in the process of assessing mental capacity. This approach, novel and rarely considered in depth from a bioethical perspective, is what we aim to develop in this article. This integration would not only enrich the MacCAT-T but also provide a more complete and humane assessment of the patient’s mental capacity. In this way, the risk of paternalistic decisions can be reduced, ensuring that evaluations better reflect the reality of the patient’s decision-making process (Fleisje 2024). This would be especially important in the context of euthanasia in patients with psychiatric pathology.

In the following section, we will frame the assessment of mental capacity within a proposal for a care relationship that incorporates the patient and their context, thus fostering a more humane and empathetic doctor-patient relationship. The assessment of mental capacity cannot be complete without considering the process of shared decision-making, a fundamental aspect in the context of euthanasia.

## The importance of shared decision-making in the euthanasia process

Organic Law 3/2021 details how the entire process of assisted dying should unfold, from the initial request by the patient to the application of euthanasia (Organic Law 3/2021, arts. 8–12). In the initial phase, when the patient requests help to die, “a deliberative process begins regarding their diagnosis, therapeutic possibilities, and expected outcomes, as well as potential palliative care, ensuring that they understand the information provided” (Organic Law 3/2021, art. 8.1). It is at this stage that the assessment of mental capacity should be integrated, specifically within a shared decision-making process.

Shared decision-making is a communication model in the health field that promotes collaboration between patients and healthcare professionals to decide on treatments and care. This model is based on a collaborative relationship characterized by respect and empathy. It assumes that patients have the right to be informed and to actively participate in decisions affecting their health. This implies a bidirectional exchange of information in which healthcare professionals provide clinical data, and patients share their values, preferences, and personal circumstances. This deliberative process aims to reach consensual decisions that reflect both the best available scientific evidence and the patient’s preferences and values (Aoki 2020). Undoubtedly, all of this requires a hermeneutic, deliberative, and empathetic task by the healthcare professional.

In any case, shared decision-making offers significant benefits: it increases patient participation and commitment to their treatments, making them feel more empowered. It also reduces decision-making conflict and increases trust in the doctor-patient relationship, translating into better clinical outcomes (Aoki et al. 2022).

This type of care relationship promotes respect for patient autonomy and ensures that decisions are made informatively and with a complete understanding of all implications. This is especially relevant in the context of euthanasia, where the decision has irrevocable consequences. It is essential that the patient receives all relevant information, including all therapeutic options and access to palliative care. Furthermore, it is crucial to assess the patient’s values, ethical principles, and life history in the context of their request for help to die.

Therefore, the assessment of mental capacity must be integrated into this broad deliberative process and not limited to a merely functional evaluation that considers only cognitive aspects.

Additionally, a hermeneutic and narrative perspective allows for a more rigorous understanding of the patient’s euthanasia request. Those responsible for assessing mental capacity must have an ethical and empathetic commitment to ensure this entire process.

## The role of empathy in the assessment of mental capacity

In healthcare, empathy plays a crucial role in the quality of care, as patients want doctors to be interested in them and take care of them. In this sense, empathy seems to contribute to this, although it should not be idealized and thought to always contribute positively to patient care (Elodie Malbois & S. HurstMajno, 2023). The literature highlights the need for a more nuanced and relational understanding of empathy, which integrates both affective and cognitive aspects and is grounded in patient-centered ethical principles and values (Sandra H., et al., 2016; Hardy 2019; Lee et al. 2022).

Five key elements of empathy have been identified (Elodie Malbois & S. HurstMajno, 2023, Mark A. Graber, John W. Ely, 2018). First, perspective-taking, which involves imagining the situation from the other’s point of view. Second, affective empathy, which is the ability to experience emotions that reflect the emotional state of the other. Third, emotional contagion, which involves automatically and unconsciously feeling the emotions of others. Fourth, empathic concern, which is an emotional reaction of care and concern for the well-being of the other. And finally, empathic distress, an aversive self-oriented reaction to the suffering of another.

Empathy in medical practice must be approached from a dialogical and narrative perspective, imbued with ethical principles and patient-centered values. This dialogical perspective considers empathy as a relational process where communication and narrative play a decisive role in mutual understanding and connection between doctor and patient. Sometimes, this empathy is characterized as the “golden rule,” which can be in negative or positive form. In the first case, it would be “do not do to others what they do not want done to them.” In the second case, it would be “treat others as they wish to be treated.”

In the assessment of mental capacity, empathy is fundamental. Putting oneself in the other’s shoes is an ethical recognition that transcends clinical judgment. It allows the healthcare professional to appreciate the patient’s suffering, understand their internal experiences, and assess their life story and circumstances. Thus, empathy becomes a moral virtue that facilitates a comprehensive understanding of the patient as a human being. Recognizing their feelings and values is essential for a fair and thorough assessment of their reasoning in requesting euthanasia. Professionals need great empathic skills to understand the ethical motives behind the request for help to die, the unbearable suffering, and the impact of psychopathology on daily life. This does not necessarily imply approving euthanasia but being willing to seriously consider the patient’s wishes and assess their capacity to make informed decisions about their life without stigma or prejudice.

Therefore, it is necessary to articulate the assessment of mental capacity with an ethical foundation based on narrative and hermeneutic ethics, along with a dialogical position. Empathy in this ethical context not only facilitates understanding the patient’s reality but also promotes a space for dialogue to explore the reasons behind their decisions. Thus, the assessment of mental capacity must be integrated into a broad deliberative process and not limited to a merely functional evaluation that considers only cognitive aspects. Assessors must have an ethical and empathic commitment, deeply understand the patient’s values, desires, and preferences, and comprehend the suffering they are going through. Additionally, they must strive for the full development of human rights and the vindication of human dignity.

In the next section, we will present, without aiming to be exhaustive, a theoretical framework grounded in ethical principles and supported by ethical theories. This would help to rigorously underpin the assessment of mental capacity of people requesting euthanasia for mental health reasons.

## Ethical principles and narrative ethics as ethical foundations for the assessment of mental capacity

In the context of a request for euthanasia, the assessment of mental capacity is fundamental to ensure that decisions are autonomous, informed, and reflect the patient’s values and desires. This assessment must be supported by ethical justification beyond cognitive analysis. It is essential to respect the person, understand their life story, and empathize with their suffering, ensuring dignified treatment in a process where the patient is extremely vulnerable.

Vulnerability is an inherent condition of all human beings, manifesting in various forms and dimensions influenced by economic, sociocultural, political, regional, educational, intellectual, and physical factors (Mergen and Akpinar 2021). Not all people with mental health problems are vulnerable in the same way or to the same extent; therefore, each request for euthanasia must be evaluated individually to understand it thoroughly.

This vulnerability directly affects the autonomy of individuals. Autonomy is often understood from a liberal perspective, where each individual makes decisions based on their principles and values. However, a relational perspective could enrich this concept by considering interpersonal and contextual relationships and how these relationships affect personal identity (Mercer Gary, 2023). In mental health, this perspective allows for a comprehensive understanding of an individual’s life story in their particular context. People should be able to decide which options are beneficial or harmful, considering their context and the impact on their lives and those of their loved ones. This is essential to understanding the patient’s suffering and the reason for their request for euthanasia.

Healthcare professionals, based on a Hippocratic tradition, may adopt paternalistic stances that reject euthanasia. However, it must be recognized that the patient knows what is best for themselves. An empathetic attitude from the professional can lead to understanding that sometimes the best for the patient is to help them die. Empathy is essential to understanding their suffering and life story. The assessment of mental capacity must consider the desires and overall well-being of the patient, ensuring that the decision is in their best interest.

The principle of non-maleficence obliges healthcare professionals not to intentionally cause harm. In euthanasia, the assessment of mental capacity must ensure that the patient’s decision is not influenced by their mental illness, thus avoiding irreversible harm. It is also necessary to confirm that the patient has understood all medical information. It is essential to avoid paternalistic decisions and ensure that evaluations faithfully reflect the patient’s decision-making process, reducing the risk of harm.

When considering harms and benefits, people seek to achieve a certain well-being, which includes living autonomously, having meaningful personal relationships, personal achievements, understanding, aesthetic enrichment, physical and mental functioning, and enjoyment (Grazia and Millun 2021). This well-being is most affected in the unbearable suffering of the person. Deeply understanding their suffering and life story helps foster respect for their dignity and autonomy. It is not only about what they desire, but also about vindicating what is just for a dignified life and death.

Dignity is an absolute value in every human being, granting them the condition of a rights bearer. It also manifests as an attitude (Weber-Guskar 2020). In the context of a request for help to die, many people consider their life undignified. Atienza (2022) argues that the core of this principle lies in the right and obligation of each individual to develop as a person, and in the obligation of others to contribute to their free and equal development. This approach requires non-discrimination and respect for individual freedom, ensuring a domain of non-interference. A proper assessment of mental capacity seeks to foster the patient’s development as a person, exercising their autonomy and deciding about their body and life.

Narrative ethics emphasizes the importance of personal stories and individual experiences in ethical decision-making. According to Howard Brody and Mark Clark (2014), this perspective is based on hermeneutics and dialogue, where interpreting experiences and values is fundamental to understanding ethical dilemmas. This approach focuses not only on events but on layers of meaning and ethical values that require contextualized and critical interpretation. This is especially relevant in the assessment of mental capacity, as it allows appreciating the meaning of the patient’s life story and the authentic suffering of the patient requesting help to die.

The practice of narrative ethics requires specific skills (McCarthy 2003). Interpretative competence and empathy are essential for listening to and understanding patients’ personal stories. The ability to communicate effectively and sensitively is fundamental and involves active listening, asking pertinent questions, and expressing understanding and support. Recognizing and valuing each patient’s individuality, understanding that their experiences, values, and beliefs influence their perception of health and the decisions they make.

In healthcare, narrative ethics gains significant relevance by promoting patient-centered care and context. Frank (2014) highlights that this approach transforms the care relationship, allowing patients’ stories to influence decision-making and treatment. This humanizes medicine and adapts care to the emotional and psychological needs of patients. Charon (2014) emphasizes narrative reciprocity as a means to achieve more equitable and just healthcare.

McCarthy suggests that narrative ethics perfectly complements principlism (McCarthy 2003). While principlism is based on universal ethical principles, narrative ethics adds an additional dimension by considering patients’ individual stories and contexts.

In cases of euthanasia in patients with mental illnesses, narrative ethics offers a solid foundation for more humanely assessing the patient’s mental capacity. Incorporating personal stories into medical practice improves decision-making and ensures more empathetic care. Thus, the assessment of mental capacity becomes more holistic and respectful, integrating emotional and value aspects instead of focusing solely on the functional and cognitive. This allows for a deeper understanding of suffering and the desire to die, valuing the patient’s autonomy and dignity more justly. It is within this ethical claim that we frame the need to introduce the medical ethics expert.

## The role of the medical ethics expert in the guarantee commissions

Organic Law 3/2021 establishes the creation of Guarantee and Evaluation Commissions to verify and control the compliance with the law. Spain is composed of autonomous communities, similar to how the United States is composed of states. Each autonomous community in Spain will have one of these commissions, which will be multidisciplinary and consist of at least seven members, including doctors, nurses, and jurists (Organic Law 3/2021, art. 7).

In Spain, the Guarantee and Evaluation Commissions overseeing the implementation of Organic Law 3/2021 on euthanasia are composed of jurists, doctors, and nurses. This composition allows for a legal and clinical approach. However, in our view, greater participation by ethics experts would be highly valuable, as they could enrich deliberations with philosophical and bioethical perspectives. A historical and significant example of this interdisciplinary integration is the United States President’s Commission (1983), which included philosophers to address complex issues such as the definition of death. This set a precedent for the importance of incorporating ethical reflections into the formulation of public policies related to end-of-life matters.

The main functions of the Guarantee and Evaluation Commission are (Organic Law 3/2021, art. 18): (1) addressing complaints from people who have been denied euthanasia and resolving conflicts of interest; (2) ensuring that the assistance in dying is carried out according to the law; (3) detecting problems in the law’s compliance and suggesting improvements for good practice manuals and protocols; (4) acting as a consultative body to resolve doubts about the law’s application; and (5) publishing an annual report on the law’s application.

Although the law does not specify the individual functions of the commission members, it can be deduced that each has specific roles according to their specialization due to its multidisciplinary composition. Doctors and nurses clinically evaluate the cases and validate the medical criteria, while jurists ensure that the procedures comply with the law and protect the rights of the applicants and healthcare personnel. The medical ethics expert could provide guidance to the healthcare team during the evaluation of mental capacity, helping to interpret complex ethical dilemmas that arise when the patient’s capacity is in doubt, especially in cases influenced by emotional and value factors. This could be achieved through ethical discussions with doctors and psychiatrists, guiding them on how to consider the patient’s emotions and values in the evaluation of their mental capacity.

Additionally, ethics experts analyze the ethical and narrative context of the patient, and how the patient’s values, life experiences, and personal narratives influence their decision-making capacity. This approach allows for a deeper and more nuanced understanding of the authenticity and coherence of the patient’s decisions, ensuring that the evaluation respects their dignity and personal perspective.

The medical ethics expert is important for facilitating ethical deliberation within the Commission, promoting a reflective and critical dialogue in which the moral and emotional aspects of each particular case are analyzed. This dialogue is not simply an exchange of opinions but a structured reflection in which the expert guides participants to question assumptions, examine the ethical implications of decisions, and consider the patient’s narratives in their entirety. The hermeneutic approach invites commission members to explore how the patient’s values and experiences influence their decision-making. The ethics expert fosters critical questions about autonomy, dignity, and suffering, encouraging commission members to think beyond traditional clinical and legal frameworks. This critical and empathetic perspective allows contextualizing the patient’s decision within a broader ethical framework.

Incorporating a medical ethics expert would improve the work of the commissions by providing an ethical perspective and ensuring respect for human rights. This expert is essential during ethical deliberation to ensure decisions are based on ethical principles, considering all contextual and narrative aspects of the patient, especially in the assessment of mental capacity.

Although the Guarantee and Evaluation Commissions in Spain act after the assessment of mental capacity by the responsible and consulting physicians, ethics experts can play a crucial role in reviewing whether the process was ethical and respectful of the patient’s life story, values, and context. Their relevance lies in applying approaches based on narrative, hermeneutic, and dialogical ethics to verify that the decision-making process has fully considered the patient’s perspective. It is not just a technical check but ensuring that an environment of respect, empathy, and mutual understanding has been promoted, evaluating whether the final decision truly reflects an ethical and consensual deliberation.

These experts are fundamental in addressing the ethical and human complexities in euthanasia requests. They not only provide a comprehensive ethical perspective but also complement the roles of jurists and doctors. Unlike jurists focused on the law and doctors focused on clinical aspects, medical ethics experts deeply understand the values, ethical principles, and individual contexts of patients.

An ethics expert must have competencies in ethical decision-making and moral deliberation, empathetic communication and active listening, interpretation of the patient’s narrative, holistic evaluation of clinical situations, and mediation in ethical conflicts.

In the assessment of mental capacity, an ethics expert provides a holistic perspective that considers cognitive, emotional, and ethical aspects, fostering a critical and ethical attitude among professionals. Additionally, they can mediate conflicts between the healthcare team, the patient, and their family, resolving ethical dilemmas and ensuring fair and empathetic decisions.

Unfortunately, the integration of medical ethics in euthanasia practices is limited. Therefore, it should be enhanced through the development of specific ethical protocols and the creation of reflective spaces for ethical debate. This integration would improve mutual understanding and the resolution of ethical dilemmas, promoting more conscious and respectful medical practice. This role would strengthen respect for ethical principles, ensuring dignity and autonomy, respecting the patient’s life story and its meaning. Promoting research in clinical ethics and bioethics is essential to develop better practices in assessing mental capacity.

Organic Law 3/2021 requires ongoing training in euthanasia for healthcare professionals. An ethics expert can design and deliver programs to raise awareness of ethical issues. For example, educational programs could include courses in bioethics and applied ethics, workshops and seminars on moral deliberation for ethical decision-making, or training in communication skills that promote empathy and narrative understanding.

Therefore, we believe that the participation of medical ethics experts in these commissions could help humanize the entire process of assisted dying. On one hand, it would encourage the introduction of ethical values and principles, aiding in a better understanding of the patient’s situation. On the other hand, it could help professionals develop greater sensitivity and advocate for the human rights of individuals, moving away from paternalistic positions that are discriminatory and stigmatizing. This is especially relevant when the patient suffers from a mental disorder.

## Conclusions

This article has explored the assessment of mental capacity in the context of euthanasia, proposing a holistic alternative approach to the traditional functional model. It has been demonstrated that the functional model is insufficient to capture the complexity of patients’ decisions. Incorporating approaches based on narrative, hermeneutic, and dialogical ethics allows for a more comprehensive evaluation, considering the patient’s life story, values, and context. This would improve the assessment of mental capacity and respect the patient’s dignity and autonomy.

The importance of shared decision-making has been emphasized, a model that fosters collaboration between the patient and healthcare professionals. In this way, it ensures that decisions about euthanasia are informed and consensual, reflecting both the best scientific evidence and the patient’s values and preferences. In this care context, empathy is fundamental in the assessment of mental capacity. Understanding and sharing the patient’s suffering and life story enable healthcare professionals to make more humane and ethical decisions. Empathy facilitates a deeper connection between doctor and patient, promoting an environment of mutual respect and understanding, which is essential for a fair and thorough evaluation.

To provide greater conceptual rigor, narrative ethics has been proposed as an ethical foundation. This approach integrates the patient’s personal experiences and values, providing a richer and more contextualized evaluation. Narrative ethics, along with ethical principles, offers a solid framework to ensure that decisions about euthanasia in the mental health field are morally just and respectful.

Finally, the need to include medical ethics experts in the Guarantee and Evaluation Commissions has been argued. These experts can bring a comprehensive ethical perspective, essential for addressing the moral and emotional complexities of euthanasia requests. Their participation ensures that decisions not only meet legal requirements but also respect the patient’s dignity and autonomy. Moreover, these experts could significantly contribute to training professionals in applied ethics or in developing ethical protocols, thus promoting clinical practices that are more respectful of individuals.

## Data Availability

No datasets were generated or analysed during the current study.
